# Intravitreal Melphalan for Vitreous Seeds: Initial Experience in China

**DOI:** 10.1155/2016/4387286

**Published:** 2016-02-08

**Authors:** Xunda Ji, Peiyan Hua, Jing Li, Jiakai Li, Junyang Zhao, Peiquan Zhao

**Affiliations:** ^1^Department of Ophthalmology, Xinhua Hospital, Shanghai Jiao Tong University School of Medicine, 1665 Kongjiang Road, Shanghai 200092, China; ^2^Department of Ophthalmology, Beijing Children's Hospital, Capital Medical University, 56 Nanlishi Road, Xicheng District, Beijing 100045, China

## Abstract

*Purpose*. To evaluate the efficacy of intravitreal melphalan for vitreous seeds from retinoblastoma in Chinese patients.* Methods*. This is a retrospective review of 17 consecutive Chinese patients (19 eyes) with viable vitreous seeds from retinoblastoma. The patients received multiple intravitreal injections of 20 ug melphalan.* Results*. The International Classification of Retinoblastoma groups were B in 1 eye, C in 5 eyes, D in 11 eyes, and E in 2 eyes. On average, 6 injections (range: 1–15) were given to each eye at the interval of 2–4 weeks. Successful control of vitreous seeds was achieved in 16 of 19 eyes (84.21%). Globe retention was achieved in 73.68% (14/19) eyes. The patients were followed up for 27 months on average (median: 26; range: 17–42 months). There is a significant difference in response to intravitreal melphalan for cloud, spheres, and dust seeds with a median number of injections of 9, 6, and 3, respectively (*P* = 0.003). Complications related to intravitreal melphalan included vitreous hemorrhage, cataract, salt-and-pepper retinopathy, and pupil posterior synechia. There was no case of epibulbar extension or systemic metastasis within the period of follow-up.* Conclusion*. Intravitreal melphalan achieved a high local control rate for vitreous seeds without extraocular extension and with acceptable toxicity in Chinese retinoblastoma patients.

## 1. Introduction

Retinoblastoma is the most common primary intraocular malignancy of infancy and childhood, with an incidence of 1 per 15,000 to 1 per 20,000 live births [1]. Intravenous chemotherapy (IVC) is an effective way of treating the disease when there is no vitreous or subretinal seeding. According to the International Classification of Retinoblastoma, the success rate of IVC combined with local therapy was 90% to 100% for groups A, B, and C; 47% for group D; and 25% for group E retinoblastoma, though these articles rarely stated the percent of D or E eyes that were primarily enucleated [2, 3]. Although there was no randomized study which compared the outcome of ophthalmic artery chemosurgery (OAC) and IVC, single institution retrospective case series seemed to suggest that OAC may have a higher success rate and less systemic side effects than IVC for D or E eyes [4, 5].

Despite the dramatic increase in ocular salvage with OAC, vitreous seeding is still one of the main reasons for subsequent enucleation in treated eyes [6]. Vitreous seeds respond poorly to chemotherapeutical drugs delivered via intravenous, intra-arterial, or periocular route. More than 50 years ago, Ericson and his group reported on the intravitreal delivery of chemotherapeutical drugs targeting vitreous seeds [7]. However, this method was not employed in routine use due to the concern on possible extraocular spread of tumor cells and inconsistent successes [8]. Half a century later, several groups revisited the chemotherapeutical drugs and intravitreal drug delivery methods for treating vitreous seeds. In 2011, Suzuki and Kaneko reported the results on intravitreal delivery of melphalan to treat 237 eyes of 227 patients with vitreous seeds [9]. Only one eye (0.4%) had extraocular metastasis. These same patients also received OAC and half also received external beam irradiation. So it is difficult to know the contribution of the intravitreal injection to overall success. Other groups in Europe and America then showed that intravitreal chemotherapy with melphalan is an effective and safe modality for eliminating vitreous seeds from retinoblastoma when the dose was increased to 20–30 ug [10–12].

Here, we report our experience on intravitreal melphalan in treating vitreous seeds in 17 Chinese retinoblastoma patients.

## 2. Methods

This is a retrospective, noncomparative, interventional study. The study followed the tenets of the Declaration of Helsinki and was undertaken with the understanding of each guardian of participating patient. Informed written consent was obtained from each guardian.

### 2.1. Patients

Seventeen consecutive Chinese retinoblastoma patients with active vitreous seeds who received intravitreal injection of melphalan between November 2011 and August 2013 were included. There were 6 eyes with resistant vitreous seeds and 13 eyes with recurrent vitreous seeds after completion of 2-3 sessions of OAC and/or 4–6 sessions of IVC. Informed written consent was obtained from each guardian.

### 2.2. Treatment

Intravitreal injection was performed under general anesthesia. Before injection, the location of retinal tumor and vitreous seeds was identified using RetCam and indirect ophthalmoscope. In order to minimize reflux, hypotony was induced by aspirating 0.1 mL of anterior chamber fluid using a 27-gauge needle prior to intravitreal injection. Anterior chamber fluid was sent for cytopathological analysis. Scleral entry site was selected at 2.5–3.0 mm away from the limbus to avoid touching the tumor tissue, vitreous seeds, and detached retina. A 30-gauge needle attached to a tuberculin syringe was placed perpendicularly through the sclera entry. Twenty-microgram melphalan in 0.1 mL solution was injected slowly and continuously within 3 seconds. After pulling out the needle, the scleral injection site was pressed using cotton swab for about 5 seconds. Forty-microgram melphalan in 0.2 mL was administered subconjunctivally around the scleral injection site. Tobradex eye drops were used three times a day for 3 days. Retinal tumors were treated with OAC, IVC, and focal consolidation such as transpupillary thermotherapy, laser coagulation, and cryotherapy if necessary. We assessed response to treatment and complications under anesthesia every 2–4 weeks with RetCam and indirect ophthalmoscope. According to Munier's study [11], complete response is established if the seeds present completely disappear (vitreous seeds regression type 0), refringent and/or calcified residues (type I), amorphous often nonspherical inactive residues (type II), or a combination of the latter two (type III). Multiple injections were performed to control vitreous seeds. When complete response is noted, intravitreal injection would be stopped.

Data was analyzed by one-way ANOVA using Statistical Package for the Social Sciences (SPSS version 19) after Levene's test for the equity of variance. *P* value equal to or less than 0.05 was considered statistically significant.

## 3. Results

Nine patients with bilateral and 8 with unilateral retinoblastoma (10 boys and 7 girls) were included in the study. The median age at diagnosis was 19 months (range 8–73 months). Based on the International Classification of Retinoblastoma, these eyes were classified as group B (*n* = 1), C (*n* = 5), D (*n* = 11), or E (*n* = 2). A total of 19 eyes were treated, which included 11 eyes (58%) with localised vitreous seeds (confined to one quadrant) and 8 (42%) with extensive vitreous seeds (more than one quadrant). Based on morphologic features, vitreous seeds were classified as dust (*n* = 5), spheres (*n* = 8), and cloud (*n* = 6) [12]. The median age at the first injection was 27 months (range 11–109 months).

**Figure 1:**
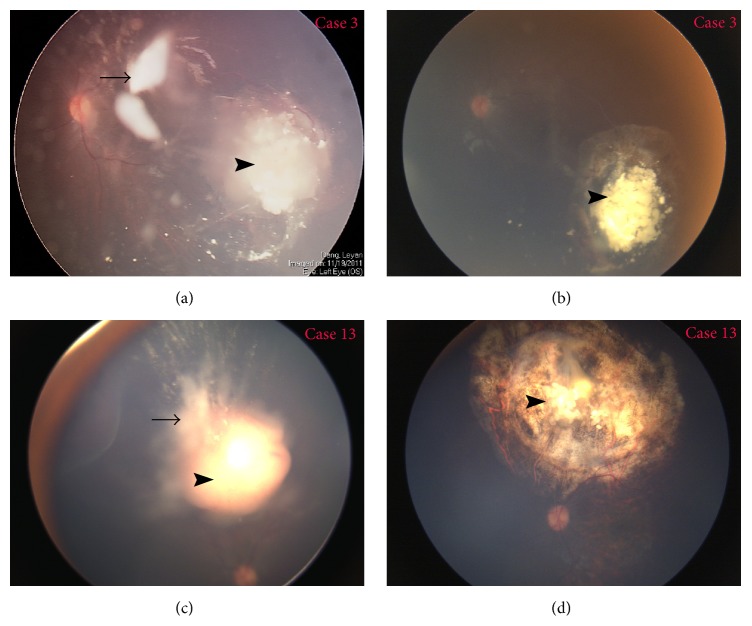
Control of resistant seeds in case  3 (upper) and case  13 (lower). (a) and (c) Vitreous seeds (arrow) and tumor (arrow head) were still active after 4 cycles of IVC and focal consolidation in case  3 and 4 cycles of IVC and 2 cycles of OAC in case  13. (b) After 11 cycles of intravitreal melphalan, 2 additional IVC, and focal consolidation, vitreous seeds regressed into type 1 and tumor (arrow head) shrank and completely calcified. (d) After 4 cycles of intravitreal melphalan, one additional OAC, and focal consolidation, vitreous seeds regressed into type 0 and tumor (arrow head) shrank and completely calcified.

**Figure 2:**
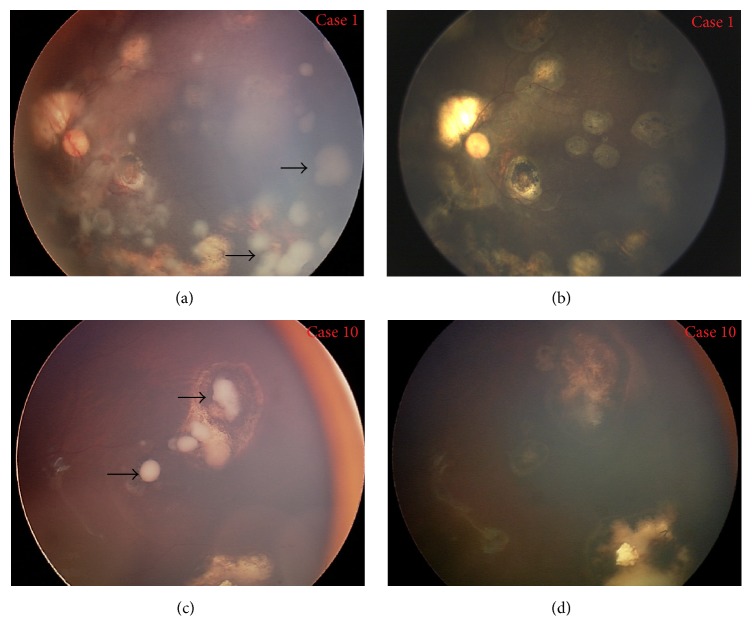
Control of recurrent seeds in case  1 (upper) and case  10 (lower). (a) Extensive spheres vitreous seeds (arrow) were noted after 6 IVC, 1 OAC, and focal consolidation in case  1. (c) Localised spheres vitreous seeds (arrow) were noted after 5 IVC and focal consolidation in case  10. (b) and (d) After intravitreal melphalan, vitreous seeds in both cases regressed into type 0.

The detailed information of each treated patient was listed in Table 1. In total, these cases received 123 (median, 6 times; range, 1–15 times) intravitreal injections delivered every 2–4 weeks. Two patients received bilateral injection and others received unilateral treatment. In this study, vitreous seeds in all cases regressed after the completion of intravitreal injection with type 0 (*n* = 10), type I (*n* = 3), and type III (*n* = 6) (Figures 1 and 2). There is a significant difference in response to intravitreal melphalan for cloud, spheres, and dust seeds with a median number of injections of 9, 6, and 3, respectively (*P* = 0.003). No significant difference was noted in the number of injections to control diffuse and localised seeds, and between recurrent and resistant seeds.

Overall, vitreous seeds were successfully controlled in 16 out of 19 eyes (84.21%). Extensive recurrence of vitreous seeds was found in 3 eyes which resulted in enucleation. The interval between the end of intravitreal injection and recurrence was 2, 3, and 7 months, respectively. No recurrence of retinal tumor was noted in the 3 eyes. Except for the 3 eyes, 2 more eyes were removed due to retinal tumor recurrence (*n* = 1) and hypotony after vitrectomy (*n* = 1). Globe retention was achieved in 14/19 (73.68%). The patients were followed up for 27 months on average (median: 26; range: 17–42 months). Cytopathological examination of the anterior chamber fluid was negative for malignant cells in each case.

Mild vitreous hemorrhage developed and cleared within 2 months in 2 cases. Cataract was noted in 3 cases, 1 of which received cataract surgery. A localised peripheral salt-and-pepper retinopathy was found in 8 eyes near the site of injection. Pupil posterior synechia was noted in 1 case. No occurrence of endophthalmitis or rhegmatogenous retinal detachment was noted. There was no case of extraocular extension or metastasis within the period of follow-up.

## 4. Discussion

This study summarises our experience performing intravitreal injection of melphalan to treat vitreous seeds from retinoblastoma. It showed that intravitreal injection with melphalan could achieve high control rate with 84% (16/19) for vitreous seeds. This is close to what was reported by other groups. Munier et al. showed an unprecedented success rate of tumor control in the presence of vitreous seeds with intravitreal melphalan [13]. Globe retention was achieved in 87% (20/23) of their case series. None of the treated eyes required EBRT to control vitreous seeds. According to the work of Japanese group, 68% of eyes treated with intravitreal melphalan achieved complete vitreous seed remission in the long follow-up [14].

In this study, although 3 patients (cases  3, 14, and 9) received additional IVC and/or OAC during the period of intravitreal melphalan, we think that regression of vitreous seeds in these cases was mainly due to the intravitreal melphalan. Case  3 received 4 IVC and case  13 received 4 IVC and 2 OAC before intravitreal melphalan. But there were still cloud vitreous seeds. So IVC and OAC had minimal effect on vitreous seeds in the 2 cases. Case  9 received additional 1 IVC and intravitreal melphalan for vitreous seeds, but the treatments failed to control vitreous seeds that resulted in enucleation.

Francis et al. found that eyes with dust seeds received fewer injections and a lower cumulative dose of melphalan, whereas eyes with clouds seeds received more injections and a higher cumulative dose of melphalan [12]. In this study, we also found that eyes with dust seeds have the best response to intravitreal melphalan, while eyes with cloud seeds have the worst response. Recent reports by Ghassemi et al. showed that the combination of intravitreal melphalan and topotecan injection was effective for refractory vitreous seeds from retinoblastoma [15]. Complete control of vitreous seeds was achieved in all 9 eyes. So combination of multiple chemotherapeutical agents may be needed to maximize the therapeutical power for cloud vitreous seeds.

One of the major concerns regarding intravitreal injection for vitreous seeds was the risk of having cancer cell spread extraocularly. Different techniques have been employed to minimize the risk, such as the employment of repetitive freeze and thaw cycles at the injection site when pulling out needle [11]. Francis et al. pointed out that irrigation with sterile distilled water submersion on the surface of the eye for at least 3 minutes could further reduce the risk in addition to freeze-thaw cryotherapy [16]. We took the following measures: (1) choosing the entry site far away from tumor and vitreous seeds, (2) performing paracentesis to soften the globe before injection to prevent retroflex of intraocular fluid when pulling out the needle, (3) pressing the scleral injection site for about 5 seconds after retracting the needle, and (4) injecting 40 ug of melphalan in 0.2 mL subconjunctivally around the scleral injection site. The absence of metastasis in our cases suggested that these precautions may be effective.

The dose of melphalan for intravitreal injection was another important issue. In this study, we used the dose of 20 ug in order to minimize the damage to the retina. No severe complications related to intravitreal melphalan were observed in our study. Ghassemi and Shields reported that 50 ug melphalan could lead to the severe complications such as subretinal hemorrhage, severe hypotonia, and phthisis [17]. In another study, Ghassemi et al. used 40 ug melphalan [15]. The results showed no changes in the a and b waves of bright-flash electroretinograms. However, it is generally accepted that melphalan of less than 30 ug is safer for the retina [6]. Except for electroretinograms, other various modalities such as vision acuity test, visual evoked potentials, fluorescein angiography, and optic coherence tomography should be included to evaluate the safety dose of melphalan injection.

In summary, our study confirmed that the intravitreal delivery of melphalan is both an effective and a safe approach in controlling vitreous seeds from retinoblastoma in Chinese patients.

## Figures and Tables

**Table 1:** Characteristics and treatment details of each treated patient.

Patient	Eye treated	Group	Previous treatment	VS type before injection	VS regression type	Times of injection	Complication	Additional treatment	Follow-up (mo)
1	OS	D	6IVC 1OAC Focal	Recurrent	0	7	Severe CC SPR	Cataract surgery	29
				Extensive					
				Spheres					

2	OS	D	4IVC 1OAC	Recurrent	0	6	SPR	NA	27
				Localised					
				Spheres					

3	OS	D	4IVC Focal	Resistant	1	11	Mild VH	2IVC Focal	36
				Extensive					
				Cloud					

4	OD	D	5IVC	Recurrent	3	6	None	Enu (VS recurrence)	3
				Extensive					
				Spheres					

5	OD	D	4IVC 1OAC	Recurrent	3	4	SPR	NA	29
				Extensive					
				Dust					

6	OS	D	6IVC	Recurrent	0	6	None	Focal	42
				Localised					
				Spheres					

7	OD	E	6IVC 2OAC	Resistant	3	10	None	Focal	34
				Extensive					
				Cloud					

8	OD	C	3IVC 6OAC	Recurrent	0	6	SPR	NA	29
				Localised					
				Spheres					

9	OS	D	6IVC	Recurrent	3	6	None	1IVC Enu (VS recurrence)	2
				Extensive					
				Spheres					

10	OS	B	5IVC Focal	Recurrent	0	6	SPR	Focal	25
				Localised					
				Spheres					

11	OS	C	5IVC Focal	Resistant	0	7	None	Focal	22
				Localised					
				Spheres					

12	OS	C	8IVC Focal	Resistant	1	8	Mild CC	Focal	24
				Localised					
				Cloud					

13	OS	D	4IVC 2OAC	Resistant	0	4	None	1OAC Focal	23
				Extensive					
				Cloud					

14	OS	D	EBRT 5IVC	Recurrent	3	15	Mild VH SPR	Enu (tumor recurrence)	2
				Localised					
				Cloud					

15	OD	D	2IVC 2OAC	Resistant	0	1	None	Focal	20
				Localised					
				Dust					

16	OD	C		Recurrent	0	4	Mild CC PPS	Focal	26
				Localised					
			4IVC	Dust					
	OS	E		Recurrent	1	5	SPR	Vitrectomy Enu (hypotony)	9
				Extensive					
				Dust					

17	OD	D		Recurrent	0	3	SPR	Enu (VS recurrence)	7
				Localised					
			6IVC	Dust					
	OS	C	2OAC	Recurrent	3	8	None	Plaque	17
				Localised					
				Cloud					

Enu: enucleation; CC: cortex cataract; VH: vitreous hemorrhage; VS: vitreous seeds; PPS: pupil posterior synechia; SPR: salt-and-pepper retinopathy; NA: not applicable.

## References

[1] Aerts I., Lumbroso-Le Rouic L., Gauthier-Villars M., Brisse H., Doz F., Desjardins L. (2006). Retinoblastoma. *Orphanet Journal of Rare Diseases*.

[2] Shields C. L., Mashayekhi A., Au A. K. (2006). The international classification of retinoblastoma predicts chemoreduction success. *Ophthalmology*.

[3] Shields C. L., Ramasubramanian A., Thangappan A. (2009). Chemoreduction for group E retinoblastoma: comparison of chemoreduction alone versus chemoreduction plus low-dose external radiotherapy in 76 eyes. *Ophthalmology*.

[4] Gobin Y. P., Dunkel I. J., Marr B. P., Brodie S. E., Abramson D. H. (2011). Intra-arterial chemotherapy for the management of retinoblastoma four-year experience. *Archives of Ophthalmology*.

[5] Schaiquevich P., Ceciliano A., Millan N. (2013). Intra-arterial chemotherapy is more effective than sequential periocular and intravenous chemotherapy as salvage treatment for relapsed retinoblastoma. *Pediatric Blood and Cancer*.

[6] Francis J. H., Schaiquevich P., Buitrago E. (2014). Local and systemic toxicity of intravitreal melphalan for vitreous seeding in retinoblastoma: a preclinical and clinical study. *Ophthalmology*.

[7] Ericson L. A., Rosengren B. H. (1961). Present therapeutic resources in retinoblastoma. *Acta Ophthalmologica*.

[8] Karcioglu Z. A. (2002). Fine needle aspiration biopsy (FNAB) for retinoblastoma. *Retina*.

[9] Suzuki S., Kaneko A. Vitreous injection therapy of melphalan for retinoblastoma.

[10] Tuncer S., Balci Ö., Tanyildiz B., Kebudi R., Shields C. L. (2015). Intravitreal lower-dose (20 *μ*g) melphalan for persistent or recurrent retinoblastoma vitreous seeds. *Ophthalmic Surgery, Lasers and Imaging Retina*.

[11] Munier F. L., Gaillard M.-C., Balmer A., Beck-Popovic M. (2013). Intravitreal chemotherapy for vitreous seeding in retinoblastoma: recent advances and perspectives. *Saudi Journal of Ophthalmology*.

[12] Francis J. H., Abramson D. H., Gaillard M., Marr B. P., Beck-Popovic M., Munier F. L. (2015). The classification of vitreous seeds in retinoblastoma and response to intravitreal melphalan. *Ophthalmology*.

[13] Munier F. L., Gaillard M. C., Balmer A. (2012). Intravitreal chemotherapy for vitreous disease in retinoblastoma revisited: from prohibition to conditional indications. *British Journal of Ophthalmology*.

[14] Suzuki S., Aihara Y., Fujiwara M., Sano S., Kaneko A. (2015). Intravitreal injection of melphalan for intraocular retinoblastoma. *Japanese Journal of Ophthalmology*.

[15] Ghassemi F., Shields C. L., Ghadimi H., Khodabandeh A., Roohipoor R. (2014). Combined intravitreal melphalan and topotecan for refractory or recurrent vitreous seeding from retinoblastoma. *JAMA Ophthalmology*.

[16] Francis J. H., Xu X. L., Gobin Y. P., Marr B. P., Brodie S. E., Abramson D. H. (2014). Death by water: precautionary water submersion for intravitreal injection of retinoblastoma eyes. *The Open Ophthalmology Journal*.

[17] Ghassemi F., Shields C. L. (2012). Intravitreal melphalan for refractory or recurrent vitreous seeding from retinoblastoma. *Archives of Ophthalmology*.

